# Iodide of Potassium in the Treatment of Typhoid Fever

**Published:** 1884-01-20

**Authors:** 


### IODIDE OF POTASSIUM IN THE TREATMENT OF TYPHOID FEVER.

Dr. R. R. Barbour writes (Louisville Medical News) that he has found vast success in the above disease by the following treatment: When the diarrhea was not well marked, I gave castor-oil and glycerine, aa ℥ iv, to relieve the bowels of all ingesta and irritative secretions; after this v gr. of potassium iodide were given every four hours, largely diluted with water. Between the doses of the potassium, four drops of the oil of turpentine in mucilage were also given. When diarrhea was persistent, five drops tincture of opium were added to each dose of the turpentine until the diarrhea was checked. The bowels were rubbed three times a day with equal parts of olive oil and turpentine, and a flaxseed poultice was kept on the abdomen as long as tenderness remained. The extremities and body, when dry and hot, were sponged frequently. I required the bowels to be moved every thirty hours, to prevent the accumulation of acrid secretions. The diet was strictly fluid, and in small quantities at a time. Buttermilk was substituted for water, if the patient preferred it. This has generally agreed well with the stomach, and has produced no diarrhea. On the sixth or seventh day of treatment the skin becomes gently moist, and on the following day the temperature begins to decline, and to continue to do so. The temperature is normal on the twelfth day of treatment. The tongue continues moist throughout the continuance of the disease.

I claim that this treatment arrests the congestion and inflammation in the first stage of the fever, being the ulcerative stage prevented.

There has been no relapse in any of the above cases, and no other remedies but the potassium and turpentine were used, neither quinine nor stimulants being given.

